# Bridging the age gap: a review of molecularly informed treatments for glioma in adolescents and young adults

**DOI:** 10.3389/fonc.2023.1254645

**Published:** 2023-09-13

**Authors:** Annette Weiser, Astrid Sanchez Bergman, Charbel Machaalani, Julie Bennett, Patrick Roth, Regina R. Reimann, Javad Nazarian, Ana S. Guerreiro Stucklin

**Affiliations:** ^1^Translational Brain Tumor Research Group, Children’s Research Center, University Children’s Hospital Zurich, Zurich, Switzerland; ^2^Division of Oncology, University Children’s Hospital Zurich, Zurich, Switzerland; ^3^Division of Haematology/Oncology, The Hospital for Sick Children, Toronto, ON, Canada; ^4^Division of Medical Oncology and Hematology, Princess Margaret Cancer Centre, Toronto, ON, Canada; ^5^Department of Neurology, University Hospital Zurich and University of Zurich, Zurich, Switzerland; ^6^Institute of Neuropathology, University Hospital Zurich, Zurich, Switzerland; ^7^Department of Pediatrics, Diffuse Midline Glioma (DMG) / Diffuse Intrinsic Pontine Glioma (DIPG) Center, Children’s Research Center, University Children’s Hospital Zurich, Zurich, Switzerland; ^8^Research Center for Genetic Medicine, Children's National Hospital, Washington, DC, United States

**Keywords:** gliomas, AYA (adolescents and young adults), WHO CNS5, targeted therapy, BRAF, histone mutations, PI3K-AKT pathway, IDH mutation

## Abstract

Gliomas are the most common primary central nervous system (CNS) tumors and a major cause of cancer-related mortality in children (age <15 years), adolescents and young adults (AYA, ages 15–39 years), and adults (age >39 years). Molecular pathology has helped enhance the characterization of these tumors, revealing a heterogeneous and ever more complex group of malignancies. Recent molecular analyses have led to an increased appreciation of common genomic alterations prevalent across all ages. The 2021 World Health Organization (WHO) CNS tumor classification, 5th edition (WHO CNS5) brings forward a nomenclature distinguishing “pediatric-type” and “adult-type” gliomas. The spectrum of gliomas in AYA comprises both “pediatric-like” and “adult-like” tumor entities but remains ill-defined. With fragmentation of clinical management between pediatric and adult centers, AYAs face challenges related to gaps in medical care, lower rates of enrollment in clinical trials and additional psychosocial and economic challenges. This calls for a rethinking of diagnostic and therapeutic approaches, to improve access to appropriate testing and potentially beneficial treatments to patients of all ages.

## Introduction

Gliomas are the most common primary central nervous system (CNS) tumors across all ages (1, 2). The overall incidence rate of gliomas is estimated at 5.81 per 100,000 and is approximately three times higher in older adults compared to young children. In the adolescent and young adult (AYA, ages 15-39 years) group, gliomas constitute 29–35% of all CNS tumors with an incidence of 3.41 per 100,000 (3–5). Gliomas remain a global challenge and improving treatment strategies to reduce mortality and morbidity is a top priority in neuro-oncology. AYA patients are especially vulnerable, and gliomas represent a major cause of cancer-related mortality in this age group. Gains in overall survival rates of AYA patients after cancer diagnosis have been marginal over the last decades, especially for AYAs with CNS tumors compared with other tumor types (6), with some reports suggesting that mortality might in fact be rising for AYAs with gliomas (5, 7).

Clinical management, therapy response and outcome differ significantly between childhood and adult glioma patients. Prognosis of children diagnosed with high-grade gliomas (HGGs) is generally poor, with often limited long-term survival - months to a few years after diagnosis (8). However, prognosis for pediatric patients with low-grade gliomas (LGGs) is excellent in terms of overall survival (9), albeit being associated with high tumor- and treatment-associated morbidity (8, 10). In adults with LGG, the higher rate of malignant transformation [exceedingly rare in children (11)] leads to a poorer prognosis.

Recent advances in molecular profiling have uncovered key oncogenic drivers and distinct glioma entities. Identification of these drivers can improve diagnostic accuracy and facilitate implementation of molecularly tailored treatments. Targeting oncogenic drivers is already a cornerstone of treatment for a subset of glioma patients, most notably those with Neurofibromatosis 1 (NF1) mutations, BRAF fusions and BRAFV600E mutated LGG and HGG (12–15).

The fifth edition of the World Health Organization (WHO) CNS tumor classification (WHO CNS5), published in 2021, introduced several molecular markers to the nomenclature to improve the diagnostic accuracy of CNS tumors (16, 17). Concerning glioma classification, one of the main additions was the distinction between “pediatric-” and “adult-type” gliomas, highlighting the clinical and biological differences across age groups. Gliomas in AYA possess "pediatric-type" and "adult-type" features, but the degree of overlap and the prognostic implications of genetic alterations in AYAs remain poorly characterized (18).

Despite the significant incidence of gliomas in AYAs, they remain an understudied population with specific needs - often unmet due to gaps in clinical care and lack of research focus on this population. Even though the biological features of gliomas in pediatric and adult patients have been described, gliomas in AYA patients have not been characterized extensively yet. Further, barriers to treatment access, lower rates of enrollment in clinical trials, financial insecurities, and paucity of AYA-focused healthcare services also negatively affect the quality of care in AYAs (19, 20).

**Figure 1:**
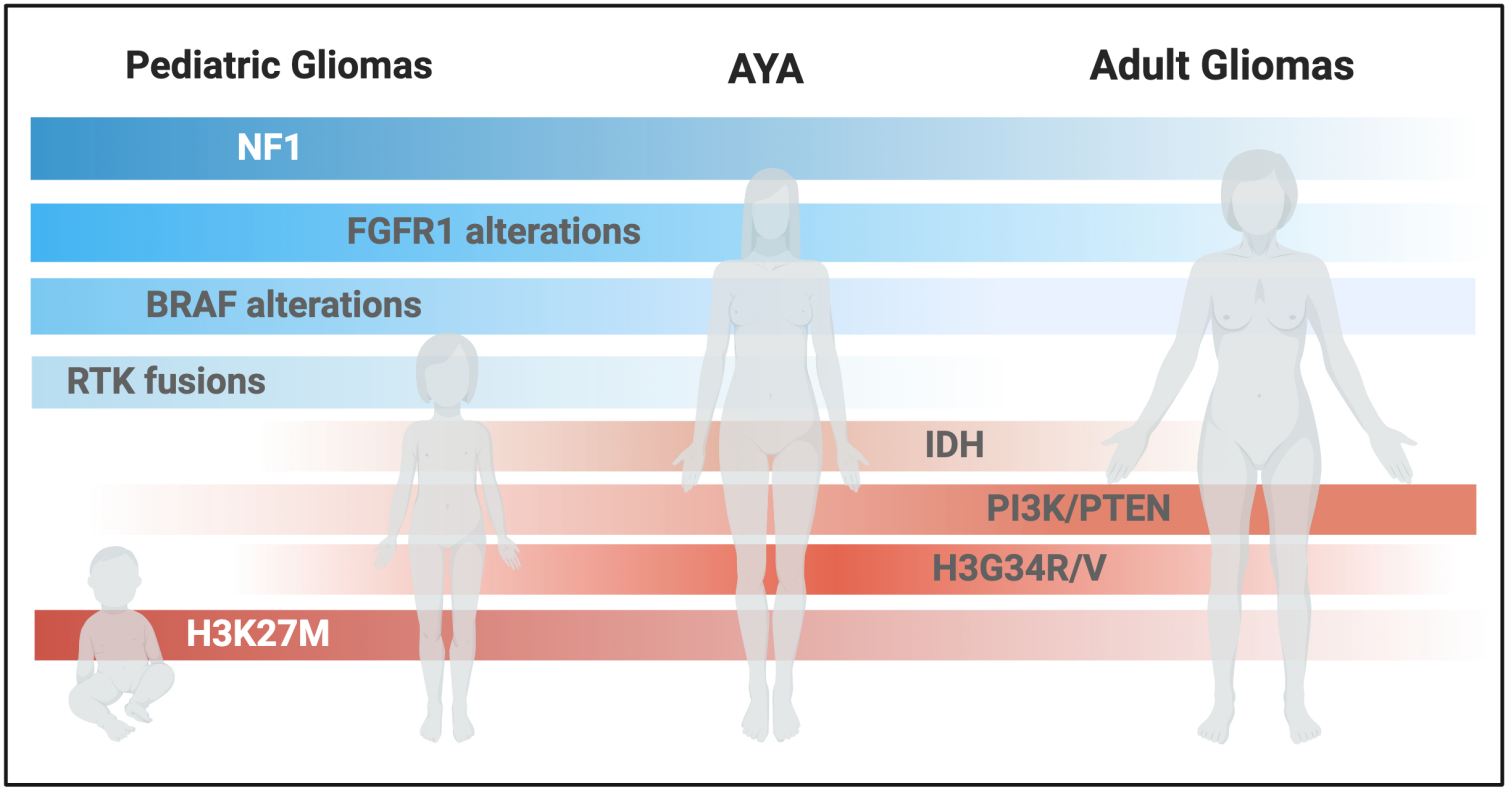
Schematic representation of glioma-associated molecular alterations across different ages. (Created with BioRender.com).

Here we review the main molecular alterations and their implications for diagnosis, prognosis, and treatment of gliomas across age groups (**Figure 1**). Highlighting current gaps in knowledge on the AYA population, we discuss targeted approaches currently under clinical investigation for patients with glioma, and potential strategies to improve access to diagnostic testing and biologically-informed treatments for AYAs.

**Figure 2:**
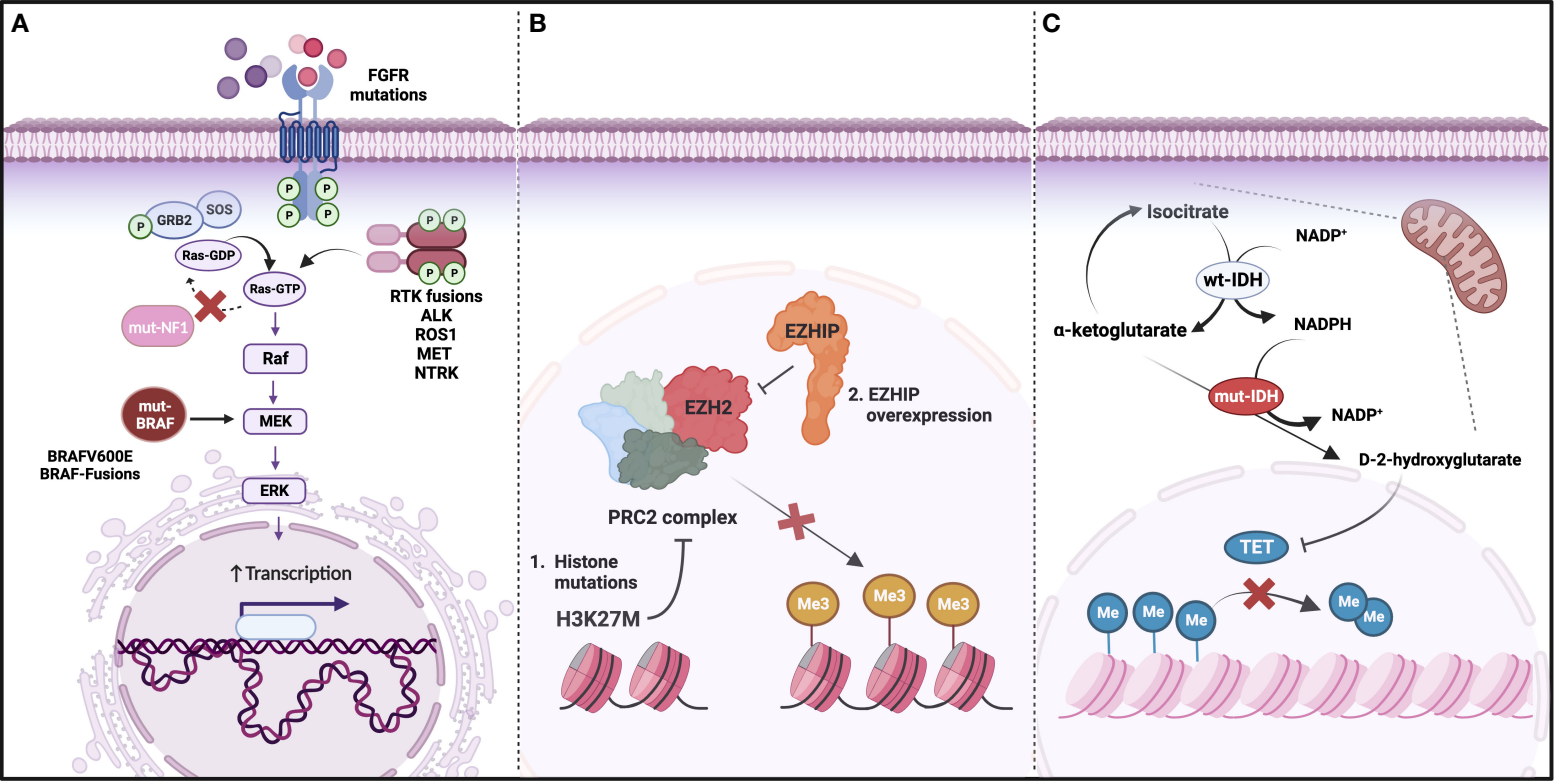
Main molecular drivers of glioma. **(A)** Genetic alterations activating the Ras/MAPK pathway, including loss of function mutations in *NF1* and gain of function mutations or fusions in *BRAF* and Receptor Tyrosine Kinases (RTKs); **(B)** DNA hypomethylation as a result of Polycomb repressive complex 2 (PRC2) inhibition by H3K27M or *EZHIP* overexpression (mutually exclusive); **(C)** IDH mutations leading to an accumulation of D-2 hydroxyglutarate and decrease in TET-mediated DNA demethylation. (Created with BioRender.com).

### Molecular features of pediatric and adult gliomas

Tumor profiling has revealed a complex glioma molecular landscape (**Figure 2**). The spectrum of genetic alterations and tumor subtypes is heterogeneous across the age continuum, with some typically diagnosed in children and others in adults (18, 21–25). Reflecting this disparity, WHO CNS5 groups gliomas into six main entities, including adult-type diffuse gliomas, pediatric-type diffuse LGG, and pediatric-type diffuse HGG. Despite this updated terminology, the patient’s age at diagnosis is not a diagnostic criterion. As such, older patients may be diagnosed with pediatric-type tumors and similarly, children may be diagnosed with adult-type tumors. While further research is needed to establish age-specific prognostic implications, we summarize how the WHO CNS5 classification highlights the biological distinctions between pediatric and adult gliomas, and potential implications for AYAs (find some details on prevalence of different types of glioma in AYAs, survival data and prognostic factors listed in **Supplemental Table S1**).

### Pediatric-type gliomas

Pediatric LGGs (PLGG) comprise a variety of histopathologic and molecular entities. Most genetic alterations underlying PLGG development are typically confined within the Ras/mitogen-activated protein kinase (MAPK) pathway, most commonly at the level of the *BRAF* oncogene (25, 26). Several molecular markers were incorporated into the WHO CNS5 classification alongside previously established histological features and immunohistochemistry information.

The glioma family of “pediatric-type diffuse low-grade glioma” includes: “diffuse astrocytoma, MYB- or MYBL1-altered”, “angiocentric glioma” (MYB::QKI fusions), “polymorphous low-grade neuroepithelial tumor of the young’’ (PLNTY, typically harboring FGFR fusions or BRAF alterations) (27), and “diffuse low-grade glioma MAPK pathway-altered” (BRAF alterations, including BRAF::KIAA1549 and BRAFV600E; and *FGFR1* alterations, including point mutations, *FGFR1* fusions and tyrosine kinase domain duplications) (16). This classification expedites diagnosis, highlighting the most common and informative molecular alterations, which should be screened for in the diagnostic workup of low-grade gliomas.

Meanwhile, HGGs are significantly less common in children compared to the adult population, where they represent the largest proportion of primary CNS tumors. HGGs are devastating diseases, associated with poor prognosis and a five-year survival below 20%, accounting for a disproportionate number of cancer-related deaths in children (28). Pediatric HGGs (PHGG) arising in midline structures of the CNS are usually driven by the somatic mutation in histones H3.1 and H3.3 encoding genes resulting in aberrant oncohistone H3K27M protein. Almost 80% of all midline PHGGs harbor H3K27M mutations while the rest exhibit overexpression of EZHIP which mimics mutant histone protein resulting in PRC2 sequestration and thus global hypomethylation (29–31). A subpopulation of PHGGs are also associated with frequent *EGFR* alterations (32, 33) which can be potential treatment targets. Tumors carrying the H3.1K27M mutation usually grow in the pons, as is the case in diffuse intrinsic pontine gliomas (DIPG); whereas H3.3K27M mutations are often identified in tumors growing in the brainstem and also other midline structures, such as the thalamus, representing diffuse midline gliomas (DMG) more generally. Interestingly, tumors with the H3.3K27M mutation are most commonly associated with brainstem location in children, whereas in AYA patients these tumors are often thalamic (18). Another common histone mutation is the H3.3G34R/V, which has been observed mostly in PHGGs of the cerebral hemispheres (23, 34), also prevalent in the AYA population (18). These primary molecularly defined entities are reflected in the WHO CNS5 “pediatric-type diffuse high-grade glioma” family, which includes “diffuse midline glioma, H3 K27-altered”, “diffuse hemispheric glioma, H3 G34-mutant,” and “diffuse pediatric-type high-grade gliomas, H3-, and *IDH*-wildtype” (16).

A rare subset of pediatric gliomas - infant-type hemispheric gliomas (IHG) - are driven by oncogenic fusions involving the receptor tyrosine kinase (RTK)-encoding genes *ALK, ROS1, MET*, and the *NTRK-*family (16, 35, 36). These fusions are common in gliomas diagnosed in very young children but have also been detected in adolescents and adults (37, 38). Though rare, these are highly targetable alterations and, in the absence of other more common alterations, should also be screened for in older patients (**Figure 2**).

### Adult-type gliomas

In contrast to pediatric-type diffuse gliomas, which are separated into LGG and HGG, this distinction is not made for adult-type gliomas (16). In adult-type diffuse gliomas - the most common malignant primary CNS tumor in adults - one main molecular feature with prognostic implications is the isocitrate dehydrogenase (*IDH)1* or *IDH2* mutation status (**Figure 2**). Adult-type diffuse gliomas are thus subdivided into “astrocytoma, *IDH*-mutant”, “oligodendroglioma, *IDH*-mutant, and 1p/19q-co-deleted”, and “glioblastoma, *IDH*-wildtype”. One important change, compared to the previous WHO CNS4 classification, is that glioblastoma is a more restricted diagnosis, encompassing diffuse and astrocytic *IDH*-wildtype tumors, typically harboring *TERT* promoter mutation and/or *EGFR* gene amplification and/or +7/−10 chromosome copy number changes. Further important molecular features implemented in the WHO CNS5 classification of gliomas include co-deletion of 1p/19q (oligodendroglioma, WHO grade 2-3), homozygous *CDKN2A/B* deletion (astrocytoma, *IDH*-mutant, WHO grade 4), as well as the presence of alterations in *MYB*, *MYBL1*, *MN1*, *YAP1*, *MYCN*, *FOXR2*, *BCOR*, *SMARCB1*, *FET-CREB*, and *DICER1* (39). In addition to the molecular classification, the presence/extent of necrosis and microvascular proliferation are still used for WHO grading (WHO grade 1-4).

#### Other gliomas

Alongside “pediatric-” and “adult-type gliomas”, the WHO CNS5 includes further glioma tumor families: “circumscribed astrocytic gliomas” (including pilocytic astrocytoma, high-grade astrocytoma with piloid features, pleomorphic xanthoastrocytoma, subependymal giant cell astrocytoma, and astroblastoma, *MN1*-altered), as well as a heterogenous group of “glioneuronal and neuronal tumors”.

### Cancer predisposition syndromes and germline testing in AYA

Cancer predisposition is another important factor to consider when evaluating AYA patients with glioma. In the pediatric population, there is a higher incidence of germline events associated with cancer predisposition. These are detected in approximately 10% of children and adolescents with cancer overall (40, 41), often with profound implications for patients and families. Though population-based data on prevalence of pathogenic germline mutations in AYAs with glioma are lacking, screening and genetic counselling should be considered, especially when family history or the presence of a somatic mutation potentially associated with cancer disposition raise suspicion for an inheritable alteration. A broad spectrum of cancer predisposition syndromes can be associated with gliomas, especially HGGs, including Li-Fraumeni syndrome (*TP53* mutation) and the germline DNA replication deficiency syndromes constitutional mismatch repair deficiency (cMMRD) syndrome and Lynch syndrome. Accurate diagnosis of constitutional mismatch repair deficiency (CMMRD)- and Lynch-associated hypermutant HGGs is critical, not only due to implications for family and tumor surveillance, but for treatment (see immunotherapy section below). For LGGs the most important cancer predisposition syndrome is NF1 leading to mainly optic pathway gliomas in 15-20% of the affected children (42).

## Biologically informed therapies

Beyond the implications for accurate tumor classification, the detection of molecular markers can facilitate access to targeted therapies. As such, appropriate molecular profiling as part of routine diagnostic testing in AYAs is the first key step, to improve the implementation of the biologically informed therapies. Several strategies targeting molecular vulnerabilities are undergoing development and optimization for glioma therapy, though typically not with a focus on AYA population. As such, in this section we review new therapeutic approaches which may be of benefit to AYA patients, despite current extensive gaps in knowledge in this population.

### BRAF/MEK inhibitors

BRAFV600E mutation and *BRAF* fusions are key drivers of pediatric LGGs (25, 43–46) and the BRAFV600E mutation is detected in a subset of pediatric and adult HGGs. With recent implementation in clinical use, BRAF and MEK inhibitors are increasingly used in treatment of pediatric and adult patients with glioma (13, 47). Vemurafenib and dabrafenib are BRAF inhibitors proven to be safe and successful in the treatment of BRAFV600E-mutated LGG in children and adults, as monotherapy, or in combination with MEK inhibitors (14, 48–50). Patients with BRAFV600E-mutated HGGs also show response to treatment with BRAF inhibitors but it is insufficient as monotherapy for cure in these patients. A randomized trial assessing the overall response rate (ORR) and tolerability of treatment with dabrafenib and trametinib versus carboplatin and vincristine in a pediatric population with BRAFV600E-mutant LGGs revealed a higher ORR, longer progression-free survival (PFS), and fewer adverse events (51). Combining MEK and BRAF inhibition also showed meaningful responses in adult BRAFV600E-mutant LGG and HGG (14).

The MEK inhibitor selumetinib has shown significant anti-tumor activity in progressive NF1-mutated and BRAF-altered PLGGs (52–54). Another MEK inhibitor, trametinib, has also been studied and proven active in patients with progressive PLGG (15, 55). Questions remain regarding optimal duration of treatment, outcomes compared with conventional chemotherapy and potential combination with other established treatment regimens. Ongoing studies are expected to answer some of these questions, including trials comparing the upfront use of selumetinib vs carboplatin/vincristine (NCT03871257), as well as a comparison of selumetinib monotherapy vs selumetinib in combination with vinblastine in patients with progressive LGGs (NCT04576117). Though designed with the pediatric population in mind, both trials allow the inclusion of young adult patients.

The pan-RAF inhibitor tovorafenib (DAY101) is being investigated in an open-label, multi-center, international phase II study (FIREFLY-1) in patients between the ages of 6 months and 25 years with BRAF-altered recurrent or progressive LGGs. The promising results from the registrational arm show an ORR of 64% with a clinical benefit rate (CBR) of 91% (56). Another ongoing trial LOGGIC/FIREFLY-2 is comparing tovorafenib monotherapy to standard of care chemotherapy in patients with PLGG harboring a RAF alteration requiring front-line systemic therapy (NCT05566795). As for FIREFLY-1, this trial also allows for inclusion of young adult patients, up to 25 years of age.

### FGFR inhibitors

Genetic alterations in *FGFR* such as point mutations or chromosomal rearrangements can occur in PLGG, whereas in adults they are more commonly detected in high-grade tumors. Emerging reports suggest that they are frequently encountered in AYA, in up to 16% of IDH-WT AYA gliomas (57). Data from this large cohort of FGFR-altered gliomas, encompassing patients aged 6 months - 87 years, fusions were more common in pediatric patients, while point mutations were more common in AYA patients. Most (87%) pediatric tumors had low-grade histology, whereas in AYA this percentage was lower (67%) and in older adult patients FGFR-altered tumors were typically high-grade. While the clinical and prognostic implications of these findings are still under investigation, this study highlights the importance of cross-age studies to uncover the landscape of molecular alterations in AYAs.

Several FGFR inhibitors have been tested in pediatric and adult patients with glioma, including erdafitinib (58) and the FGFR1–3 inhibitor infigratinib (59), which was investigated in a multicenter phase II study in patients with recurrent gliomas and FGFR alterations. Despite a low ORR of 3.8%, 4 patients had prolonged disease control (59). A pediatric study testing the oral FGFR inhibitor Debio1347 on a small cohort of 3 PLGG patients and 2 PHGG patients detected some responses (60), whereas none were observed in adult patients with HGG. Despite relatively low response rates, these early findings suggest that some patients might have durable responses to FGFR inhibition. It is likely that specific FGFR alterations and/or the presence of other concomitant alterations dictate response to FGFR inhibitors. Further studies are needed to explore these and other open questions but, given the high prevalence in AYA and positive responses in some pediatric patients, FGFR alterations should be screened for and targeted approaches considered in this patient population.

### HDAC inhibitors

Histone deacetylase (HDAC) inhibitors are increasingly used to treat H3K27M-altered DMGs and DIPGs. At a molecular level, mutated H3K27M induces an inhibition of the H3K27me3 methyltransferase complex, Polycomb repressive complex 2 (PRC2), leading to increased histone acetylation and decreased histone methylation. This global alteration of epigenetic marks results in increased expression on oncogenic programs. HDAC inhibitors have been developed with the goal to enzymatically remove histone acetyl groups from the genome under tumorigenic circumstances. One of the HDAC inhibitors under clinical evaluation for DIPGs/DMGs is panobinostat, which has also been used to treat many other cancer types. Treatment with panobinostat lead to an increase in histone acetylation, demonstrating biological activity. The therapy is generally well tolerated, despite up to 30% pediatric patients showing thrombocytopenia and anemia (61, 62). Seven children and adolescents (5-21 years) with newly diagnosed DIPG received repeat doses of convection enhanced delivery (CED) with MTX-110 (aqueous panobinostat) in the PNOC015 trial which was tolerated well. Most toxicities patients experienced were of neurological etiology. Compared with historical controls, the OS with a median of 26.1 months was encouraging but due to the limited number of participants must be interpreted with caution (63). New HDAC inhibitors are under clinical investigation to overcome the drawbacks from panobinostat, among them, quisinostat and romidepsin. Recent studies have demonstrated the efficacy of quisinostat and romidepsin in preclinical DMG models, with good BBB penetration and inhibition of tumor growth (62).

### Imipridones

Imipridones are small inhibitor molecules that have shown anti-tumor effects with promising results for several cancer treatments (64). ONC201 is a type of imipridone for which the anti-tumor effects are still being investigated and which has shown efficacy in hematological malignancies (65), as well as in H3K27M DMGs in combination with radiation (66). ONC201 was first discovered during its involvement in activating the TNF-Related Apoptosis Inducing Ligand (TRAIL)-pathway and the integrated stress response (ISR)-pathway, which are important modulators in balancing both cell survival and cell death (66, 67). ONC201 works as an antagonist for the dopamine receptors DRD2 and DRD3, both belonging to the G-protein coupled receptor family. ONC201 crosses the BBB and blocks DRD2, resulting in the activation of the ISR-pathway, TRAIL-induced apoptosis and inhibition of the AKT/ERK pathway (67). Another trial with ONC201 is ongoing for adult patients with recurrent, mainly thalamic (location in the pons or spinal cord excluded) H3K27M glioma (NCT03295396). The results of this trial will contribute to our knowledge on treating these tumors in AYA patients as H3K27M mutated gliomas are mainly located thalamic in AYAs. A new derivate of the ONC201 imipridone, ONC206, has been shown in preclinical studies (68) to be more potent than ONC201 and is currently under clinical investigation in children and young adults (up to 21 years of age) with DMG or other recurrent high-grade CNS tumors (NCT04732065). Both drugs bind to and activate the mitochondrial serine protease ClpP (caseinolytic protease proteolytic subunit), leading to mitochondrial damage, release of reactive oxygen species, activation of ISR-pathway, and apoptosis (68–70).

H3K27M DMGs are universally associated with dismal prognosis and, though affecting mostly pediatric patients, they are also prevalent in the AYA and adult population. Given the lack of curative and treatment options, there is a strong rationale for the design of age-inclusive clinical trials for DMGs.

### PI3K/mTOR inhibitors

Overactivation of the PI3K/mTOR pathway - through the presence of activating mutations (e.g. in *PIK3CA*), loss of the negative regulator *PTEN*, and/or activation of upstream receptor tyrosine kinase receptor signaling - underlies tumor growth and is a key oncogenic driver in most human cancers, including gliomas. As such, targeting the PI3K/mTOR pathway, either using a monotherapy or combinatorial approach, is a strategy that has been amply explored. The mTOR inhibitor everolimus is used to treat several CNS tumor entities. A well-known indication for therapy with everolimus is the presence of relevant, unresectable subependymal giant cell astrocytomas (SEGAs) in patients with tuberous sclerosis complex (TSC) (71, 72). Patients with TSC and associated SEGA treated with everolimus typically show a significant reduction in tumor size and a significant reduction of seizure frequency (73). Also, children with recurrent/progressive NF1-associated LGGs showed good responses to everolimus (74). Due to the known common activation of the PI3K-pathway in DIPG, everolimus was included as one of the drugs tested in the biomarker-driven platform trial BIOMEDE (NCT02233049) for children and young adults (up to 25 years of age) (75). Everolimus showed a trend towards better efficacy (not statistically significant) when compared to erlotinib and dasatinib, with a good toxicity profile.

Paxalisib is a PI3K-inhibitor under clinical investigation, which has shown encouraging responses in adult patients with recurrent HGGs (76, 77). Paxalisib is also being evaluated for safety and efficacy in HGGs, including DIPG/DMG in combination with ONC201 (NCT05009992) (78, 79).

### NTRK/ALK inhibitors

Several inhibitors have been developed targeting neurotrophic tropomyosin kinase receptors (NTRK) and/or anaplastic lymphoma kinase (ALK)-fusion proteins (80). Second-generation ALK inhibitors, such as alectinib and brigatinib have been designed with an enhanced BBB penetration to treat ALK-driven non-small cell lung cancer (NSCLC) with CNS metastasis (81). Lorlatinib, a third-generation ALK inhibitor with enhanced BBB penetration, has shown efficacy in several pediatric and adult malignancies, including in a child with ALK-fused infant-type hemispheric glioma (IHG) (82).

The first-generation TRK inhibitor larotrectinib has been approved for treatment in adult and pediatric patients with NTRK-fused CNS tumors (83). Entrectinib has also shown activity against NTRK-, ROS1-, and ALK-fused malignancies, especially in adults with NSCLC with CNS metastases. Entrectinib was approved in 2019 by the FDA to treat children >12 years old and shown to have positive anti-tumor activity both in adult and pediatric patients with NTRK- and ALK-driven CNS tumors (84, 85).

### *IDH* inhibitors

Tumor-driving isocitrate dehydrogenase (*IDH)* mutations have been identified in different types of cancer, leading to the development and implementation of several *IDH* inhibitors in clinical practice. As adult-type gliomas commonly harbor *IDH1* (and less commonly, *IDH2*) mutations, testing the efficacy of IDH inhibitors in these tumors has become a research focus in recent years.

Ivosidenib (AG-120), an *IDH1* inhibitor, was tested in *IDH*-mutant solid cancers and is being evaluated for efficacy in *IDH1*-mutant LGGs in adults. The BBB-penetrant *IDH1* inhibitor DS-1001b was evaluated in a phase I clinical trial in adult patients with *IDH1*-mutant recurrent/progressive glioma with promising results (86). Vorasidenib, an inhibitor of mutant *IDH1* and *IDH2*, was investigated in adult patients with IDH-mutant WHO grade 2 gliomas in a randomized phase III trial. Treatment with vorasidenib prolonged PFS compared to placebo-treated patients. Furthermore, the time to next therapeutic intervention was significantly longer in patients receiving vorasidenib compared to the placebo group (87).

IDH mutations are rare in the pediatric population but detected in up to 35% of glioma adolescent patients aged 14 years or older (88). This calls for a lower age of inclusion and/or AYA-focused trials (such as NCT03749187) evaluating the role of IDH inhibition in gliomas also in adolescent patients.

### EGFR inhibitors

Epidermal growth factor receptor (EGFR) gain of function, due to amplification or the presence of its active mutant EGFRvIII, is common in adult patients with HGGs, exceedingly rare in pediatric and rare in adult patients under 35 years of age (89). As such, most clinical trials developed over the last decades focused on the adult/older adult patient population. Multiple biological agents targeting EGFR, including tyrosine kinase inhibitors (e.g. gefitinib, erlotinib), monoclonal antibodies (e.g. cetuximab), antibody-drug conjugates (e.g. depatuxizumab mafodotin), as well as immunotherapeutic approaches, such as anti-tumor vaccines and EGFRvIII-specific chimeric antigen receptor (CAR) T cells, have been tested in adult glioma patients, with generally underwhelming results (90–93). The reasons for treatment failure are multifactorial and include mechanisms leading to target independence (through alteration of the structure or loss of target expression), activation of alternative signaling pathways, and limited agent distribution due to BBB’s properties (94–96).

Combination treatment of osimertinib and bevacizumab was explored in patients with tumors harboring EGFR amplification and EGFR variant III mutations but as the study cohort was small (15 patients), further evaluation is needed (97). Tesevatinib is a second-generation tyrosine kinase inhibitor that crosses the BBB and targets EGFR, human epidermal growth factor 2 (HER2)/neu, and Src, currently in phase II clinical trials (NCT02844439) (98).

### Immunotherapies

Another growing field with new treatment options for (high-grade) glioma is immunotherapy. Based on success in hematological malignancies and other solid tumors, expectations to identify immunotherapies which are effective for gliomas were built up in the past few years (34, 99, 100). Immunotherapeutic approaches include checkpoint inhibitors, cellular immunotherapy, anti-tumor vaccines and oncolytic viruses.

Drugs targeting the immunoregulatory checkpoint proteins programmed cell death protein 1 (PD1) and its ligands PD-L1 and PD-L2 and cytotoxic T-lymphocyte-associated-protein 4 (CTLA-4), which inhibit T-cell-mediated response of the patients’ immune system have been tested in clinical trials. Several of these clinical trials evaluating checkpoint inhibitors so far did not show significantly prolonged OS or PFS in pediatric and adult HGG and glioblastoma patients (101–105).

In a phase II clinical trial (Ipi-Glio trial) comparing the efficacy of ipilimumab and temozolomide versus temozolomide alone in adults with newly diagnosed glioblastoma, no difference in PFS or OS was observed (106).

The exception to this is patients with cMMRD or Lynch syndrome associated HGGs (107). These patients are unlikely to respond to temozolomide, which requires an intact MMR system for activity. After early reports suggested a benefit for patients with cMMRD-associated hypermutant HGGs treated with immune checkpoint inhibitors (108), further studies confirmed objective responses and a three-year survival of 41.4% (107). AYA patients are more likely to be diagnosed with Lynch syndrome (monoallelic germline pathogenic variants in mismatch repair genes), given that patients with cMMRD (biallelic germline pathogenic variants in MMR genes) are typically diagnosed with tumors at young age. Though Lynch syndrome-associated hypermutant tumors have a lower mutational burden compared to cMMRD-associated tumors, especially those with concomitant polymerase proofreading deficiency (genomic predictor of response to PD-1 inhibition), there are objective responses to immune checkpoint inhibitors in these patients.

CAR T cells have revolutionized treatment of refractory hematologic malignancies, but are not yet established for solid and CNS tumors (109, 110). Several targetable antigens have been identified in adult and pediatric HGG, including Ephrin-A2 (EphA2)-receptor, human epidermal growth factor receptor 2 (HER-2), B7-H3 (CD276), interleukin-13 receptor subunit α-2 (IL13Rα2), and glycolipid tumor antigen 2 (GD2) (111, 112). For both adult and pediatric HGG patients several clinical trials with different treatment strategies have been carried out and are still ongoing. Out of 16 evaluable patients (adults and children/adolescents), eight showed a clinical benefit (partial response or stable disease) to treatment with intravenous HER-2- (and pp65)-targeted CAR T cells and treatment was considered to be safe (113). Clinical trials testing HER-2-directed CAR T therapy in children with CNS tumors, EGFR-directed CARs for children and AYAs with CNS tumors and B7-H3-specific CAR Ts in patients with DIPG/DMG or refractory pediatric CNS tumors are ongoing (NCT03500991, NCT03638167 and NCT04185038). For H3K27M-altered DIPG/DMG, GD2-CAR T cells (114) and B7-H3 CAR T cells are currently under clinical investigation with promising preliminary results (115).

Vaccination has been a focus of immunotherapy research for three decades. In a randomized trial, rindopepimut, a peptide vaccine targeting EGFRvIII-positive glioblastoma in adults did not prolong survival (92). More recent developments include vaccines targeting histone H3 mutations. In a trial with patients aged 3-21 years, patients with H3.3K27M-specific CD8+ immunological responses had longer OS compared to non-responders.

Oncolytic viruses are (re-)emerging as important immunotherapeutic options, especially for pediatric and young adult patients with DIPG/DMG. Of 9/12 children with DIPG treated with the oncolytic adenovirus DNX-2401 a reduction in tumor size was documented, making this treatment another interesting development for these very high-risk tumor entities (116). On the other hand, 49 patients with recurrent glioblastoma treated with intratumoral delivery of the oncolytic DNX-2401 virus followed by intravenous pembrolizumab did not develop any dose-limiting toxicities but treatment also did not result in a statistically relevant increase of the overall response rate (117).

## Discussion

The WHO CNS5 introduced the distinction between pediatric-type and adult-type gliomas, highlighting the biological differences between tumors in these age groups. This sets the stage for further research and therapy developments, tailored to the specific needs of the pediatric and adult populations. While this will certainly be beneficial and support a focus on age-relevant research questions for those patient groups, there is a concern that AYAs will remain poorly defined, “unseen” and medically underserved.

Understanding the longitudinal overlap and glioma evolution from childhood to adulthood is an important research gap. The prevalence and prognostic impact of molecular alterations in AYA gliomas is largely unknown. While medicine in general, and oncology in particular, evolve towards biologically-informed treatment, this lack of knowledge on AYA gliomas has critical consequences. Gliomas represent a significant cause of cancer-related morbidity and mortality in AYAs and survival gains for these patients have been minimal to non-existent, with some studies suggesting that mortality might in fact be rising (5, 7).

Treatment optimization, including implementation of targeted therapies, starts with the adoption of appropriate molecular testing as part of the diagnostic work-up, for biomarker identification. Given the pediatric versus adult focus of WHO CNS5, recent consensus statements and recommendations from experts in the field are key in ensuring appropriate and timely diagnostic testing for AYA patients (118, 119).

Even though the molecular features vary between pediatric, adult, and - most likely - AYA gliomas, these tumors also share common tumorigenic pathways, including overexpression of oncogenes, activation of RTKs, epigenetic dysregulations, and increased metabolic pathways, which should be explored for introducing new therapies in age-inclusive clinical trials. As discussed above, several pediatric studies and study consortia are starting to increase the upper limit of age of inclusion, to allow enrollment of young adults with “pediatric-type” diseases, a much-needed step to increase access to innovative therapies for AYAs.

Currently, clinical management of AYA patients is highly fragmented between pediatric and adult centers, which can further limit access to therapy due to lack/disconnected information exchange between health care practitioners. To bridge this gap and offer this vulnerable group of patients better treatment options, exchange of expertise and close collaboration between pediatric and adult neuro-oncologists - and broader multidisciplinary clinical teams - is indispensable. Several centers are implementing regular joint case discussions within dedicated tumor boards, to improve the quality of care for AYA patients and increase inclusion of AYA patients in clinical trials.

Furthermore, it is important to also consider the socioeconomic and mental health burden that AYA patients experience. Due to prognostic uncertainty and treatment limitations, AYA patients report being under long-term stress due to lack of control over their future, feeling burdened, and social isolation (120). Support from specialized social workers, physical therapists and psychologists, ideally in AYA-focused treatment facilities, would contribute to advise, guide, and support AYAs during and after tumor therapy. Specialized departments also offer the possibility to connect with other patients in similar age groups, and tailored activities, such as physical activities/sports for AYA patients.

## Conclusion

There is still much to learn about gliomas in AYAs and much to do to improve clinical care and treatment. The growing awareness and identification of specific gaps in knowledge is a step in the right direction and hopefully broader changes will follow. Ensuring access to appropriate molecular testing to detect key biomarkers, designing age-inclusive clinical trials for gliomas and creating multidisciplinary teams, bridging the pediatric/adult divide, are some of the many actions needed and being implemented in several centers across the world. Further, research focusing on AYAs should be encouraged and supported, to bring new insights into tumor biology in this population.

## Author contributions

AW: Writing – original draft, Writing – review & editing. ASB: Writing – original draft, Writing – review & editing. CM: Writing – original draft, Writing – review & editing. JB: Writing – review & editing. PR: Writing – original draft, Writing – review & editing. RR: Writing – review & editing. JN: Writing – original draft, Writing – review & editing. AGS: Writing – original draft, Writing – review & editing.
