# Supplementary material for: Bridging the age gap: a review of molecularly informed treatments for glioma in adolescents and young adults
Source: Front Oncol. 2023 Sep 13;13:1254645. doi: 10.3389/fonc.2023.1254645 (PMC10533987; doi:10.3389/fonc.2023.1254645)
Supplement: Supplementary file 1 [file Table_1.docx]

Supplemental table S1: Prevalence of different types of glioma in AYAs, survival data, prognostic factors

| Type of glioma | Survival | Prognostic factors |
| --- | --- | --- |
| LGG (~2/3 of gliomas) (1) |  |  |
| - Pilocytic astrocytoma 3% (2) | - 10-year OS 80-95% (1,2,3) | **Positive prognostic factors:**   - Extent of resection / GTR (2) |
| - Oligodendroglioma 4-10% (2,4), oligodendrogliomas and oligoastrocytic tumors 3% (5) | - 5-year OS 85-92% - 10-year OS 65-90% (2) - 15-year survival probability 0.6 (5) | **Positive prognostic factors:**   - Extent of resection / GTR (2) - Age >15 years (6) |
| - Neuronal and mixed neuronal-glial tumors 3.4% (5) |  |  |
| - BRAF-fused | - 10-year OS 100% (7) |  |
| - BRAF-V600E-altered | - 10-year OS 90% (7) |  |
| - FGFR-altered | - 10-year OS 95% (7) |  |
| HGG (~1/3 of gliomas) | - Median OS 25 months (8) | **Negative prognostic factors:**   - Midline location (8) - STR (8) - H3K27-mutant (8) - Higher Ki-67 index (8) |
| - “Pediatric-type”, histone H3-mutant 40% (8) |  | **Negative prognostic factors:**   - Overexpression of p53, *TP53* mutation (2) |
| - - H3K27 26% | - Median OS 18.5 months (8) |  |
| - - H3G34-mutants 14% (8) | - Median OS 36 months (8) |  |
| - - IDH-wt (16% FGFR mutated) (9), histones’ genes-wt, with rare mutations 9% (8) |  |  |
| - “Adult-type”, IDH-mutant 28-53% (7,8) | - Median OS 56 months (8) - 10-year OS 50% (7) - 15-year OS 29% (7) |  |
| - Glioblastoma, 4.8% (5)   - 12-74% IDH negative (4,7) | - 5-year OS 18% (2) - 15-year survival probability 0.2 (5) |  |
| - BRAF-V600E-altered | - 10-year OS 14% (7) |  |
| - FGFR-altered | - 10-year OS 25% (7) |  |
| Diffuse astrocytoma 0.47/100,000 (2), diffuse astrocytic and oligodendroglial tumors 2/100,000 (5), diffuse and anaplastic astrocytoma 7.3% (5) | - 5-year OS 65-82.5 - 10-year OS 45-80% (2) - 15-year survival probability 0.5 for diffuse astrocytoma (5) - 15-year survival probability 0.4 for anaplastic astrocytoma (5) |  |

LGG = low-grade glioma; HGG = high-grade glioma; OS = overall survival; GTR = gross total resection; STR = subtotal resection, wt = wildtype

1. Malhotra AK, Karthikeyan V, Zabih V, Landry A, Bennett J, Bartels U, et al. Adolescent and young adult glioma: systematic review of demographic, disease, and treatment in"uences on survival. Neurooncol Adv (2022) 4(1):vdac168. doi: 10.1093/ noajnl/vdac168
2. Diwanji TP, Engelman A, Snider JW, Mohindra P. Epidemiology, diagnosis, and optimal management of glioma in adolescents and young adults. Adolesc Health Med Ther (2017) 8:99–113. doi: 10.2147/AHMT.S53391
3. Park JH, Jung N, Kang SJ, Kim HS, Kim E, Lee HJ, et al. Survival and prognosis of patients with Pilocytic astrocytoma: a single-center study. Brain Tumor Res Treat (2019) 7(2):92–7. doi: 10.14791/btrt.2019.7.e36
4. Vadgaonkar R, Epari S, Chinnaswamy G, Krishnatry R, Tonse R, Gupta T, et al. Distinct demographic pro!le and molecular markers of primary Cns tumor in 1873 adolescent and young adult patient population. Childs Nerv Syst (2018) 34(8):1489–95. doi: 10.1007/s00381-018-3785-y
5. Ostrom QT, Price M, Neff C, Ciof! G, Waite KA, Kruchko C, et al. Cbtrus statistical report: primary brain and other central nervous system tumors diagnosed in the United States in 2015-2019. Neuro Oncol (2022) 24(Suppl 5):v1–v95. doi: 10.1093/ neuonc/noac202
6. Matsui JK, Allen PK, Perlow HK, Johnson JM, Paulino AC, McAleer MF, et al. Prognostic factors for pediatric, adolescent, and young adult patients with non-Dipg grade 4 gliomas: a contemporary pooled institutional experience. J Neurooncol (2023) 163(3):717–26. doi: 10.1007/s11060-023-04386-4
7. Bennett J, Nobre L, Sheth J, Ryall S, Fang K, Johnson M, et al. Lgg-41. The clinical and molecular landscape of gliomas in adolescents and young adults. Neuro Oncol (2022) 24(Suppl 1):i97. doi: 10.1093/neuonc/noac079.353
8. Roux A, Pallud J, Saffroy R, Edjlali-Goujon M, Debily MA, Boddaert N, et al. High-grade gliomas in adolescents and young adults highlight histomolecular differences from their adult and pediatric counterparts. Neuro Oncol (2020) 22 (8):1190–202. doi: 10.1093/neuonc/noaa024
9. Nobre L, Sait SF, Bennet J, Larsen AG, Ho IC, Gianno F, et al. Lgg-20. Landscape of Fgfr alterations in pediatric and aya gliomas. Neuro Oncol (2023) 25(Suppl 1):i60. doi: 10.1093/neuonc/noad073.229
